# Minnesota State Veterinary Medical Association

**Published:** 1897-03

**Authors:** 


Secretary,

### MINNESOTA STATE VETERINARY MEDICAL ASSOCIATION.

THE veterinarians of Minnesota met at the Merchants' Hotel, St. Paul, January 28th, for the purpose of organizing a State veterinary medical association. A number of prominent veterinarians were present. The following officers were elected: President, Dr. S. D. Brimhall, Minneapolis; First Vice-President, Dr. J. P. Anderson, Rochester; Second Vice-President, Dr. A. Youngberg, Lake Park; Secretary, L. Hay, Faribault; Treasurer, K. J. McKenzie, Northfield.

A special meeting will be called in a short time. A regular meeting will be held at Owatonna in July, just previous to the assembly of the Southern Minnesota Veterinary and Dental Association, an organization of several years' standing, but whose work and influence have not extended to northern Minnesota.

